# Mechanisms and metabolic consequences of adipocyte progenitor replicative senescence

**DOI:** 10.1097/IN9.0000000000000046

**Published:** 2024-08-28

**Authors:** Tonghui Lin, Aftab Mohammad, Mikhail G. Kolonin, Kristin L. Eckel-Mahan

**Affiliations:** 1 The Brown Foundation Institute of Molecular Medicine, University of Texas Health Science Center at Houston, Houston, TX, USA; 2 Molecular and Translational Biology Program, MD Anderson Cancer Center/UTHealth Graduate School of Biomedical Sciences, Houston, TX, USA; 3 Department of Integrative Biology and Pharmacology, McGovern Medical School, University of Texas Health Science Center at Houston, Houston, TX, USA

**Keywords:** adipocyte progenitor cells, circadian, aging, senescence, proliferation, adipogenesis

## Abstract

In recent decades, obesity has become a worldwide epidemic. As a result, the importance of adipose tissue (AT) as a metabolically active storage depot for lipids and a key mediator of body-wide metabolism and energy balance has been increasingly recognized. Emerging from the studies of AT in metabolic disease is a recognition of the importance of the adipocyte progenitor cell (APC) population of AT being the gatekeeper of adipocyte function. APCs have the capability to self-renew and undergo adipogenesis to propagate new adipocytes capable of lipid storage, which is important for maintaining a healthy fat pad, devoid of dysfunctional lipid droplet hypertrophy, inflammation, and fibrosis, which is linked to metabolic diseases, including type 2 diabetes. Like other dividing cells, APCs are at risk for undergoing cell senescence, a state of irreversible cell proliferation arrest that occurs under a variety of stress conditions, including DNA damage and telomere attrition. APC proliferation is controlled by a variety of factors, including paracrine and endocrine factors, quality and timing of energy intake, and the circadian clock system. Therefore, alteration in any of the underlying signaling pathways resulting in excessive proliferation of APCs can lead to premature APC senescence. Better understanding of APCs senescence mechanisms will lead to new interventions extending metabolic health.

## 1. Introduction

Adipose tissue (AT) is an endocrine organ regulating energy balance, metabolism, and body weight. While the lipid-storing adipocytes make most of AT volume, the heterogeneous cells of the stromal vascular fraction include multiple cell types, including endothelial cells (ECs), immune cells, and adipocyte progenitor cells (APCs) with diverse functions. Recent work addressing mechanisms of APC proliferation and differentiation has revealed their importance in maintaining healthy AT in the context of overnutrition or aging. Cellular senescence, generally considered a state of halted proliferation, DNA damage, and presence of the so-called “senescence-associated secretory phenotype” (SASP) of APCs, has been demonstrated in fat depots throughout the body. Premature senescence of APCs predisposes to adipocyte hypertrophy, tissue dysfunction, and ultimately metabolic consequences due to the inability of AT to adequately store fatty acids. The resulting excess fat deposition in other organs over time leads to insulin resistance and the associated cardiovascular complications. This mini-review addresses several mechanisms by which APC proliferation and differentiation are controlled and factors that may lead to premature senescence, which limits healthy AT expansion through adipocyte hyperplasia and, hence, affects organism-wide metabolic health.

## 2. Progenitor cells of white adipose tissue

White adipose tissue (WAT) is distributed throughout the body in 2 major fat depots. These include the subcutaneous AT (SAT) and visceral AT (VAT). Hypertrophy of adipocytes, resulting from a limit in preadipocyte proliferation, results in hypoxia, which leads to increased cell death, inflammation, and fibrosis underlying the metabolic syndrome and type 2 diabetes ^[1]^. The process of AT changing from beige to white or vice versa is controlled by the recruitment of distinct types of heterogeneous APCs, as well as direct adipocyte conversion ^[2]^. APCs are a component of the mixed population of mesenchymal adipose stromal cells (ASCs), the fibroblastic cells of the stromal vascular fraction. ASCs reside in the perivascular niche where they also support the function of ECs ^[3]^. Depending on the cues, ASCs differentiate into fibroblasts or preadipocytes, with their different subpopulations having varying propensities for beige and white adipogenesis.

Single-cell RNA sequencing (scRNA-seq) technologies have been transformational in delineating ASC subpopulations ^[4]^. Many clusters of preadipocytes and fibroadipogenic progenitors have been shown to be at least partly conserved between mice and humans ^[5]^. Most of the studies in mice have so far focused on subcutaneous inguinal (iWAT) and perigonadal (gWAT) depots. They have revealed at least 2 major subclusters of ASCs. One subcluster consists of cells with high adipogenic potential. They express membrane tyrosine kinase platelet-derived growth factor receptors alpha (PDGFRα) and beta (PDGFRβ). The balance of transient PDGFRα/PDGFRβ expression and signaling during adipogenesis induction influences whether preadipocytes differentiate as beige or white, respectively ^[6]^. These cells are committed preadipocytes that turn on adipocyte markers such as *Pparg, Lpl,* and *CD36*. The second major group of mesenchymal stromal cells displays a gene expression signature related to extracellular matrix (ECM) remodeling and inflammation. This cell cluster has been termed fibro-inflammatory precursors and interstitial progenitor cells ^[7]^. These cells, regulating inflammation and extracellular matrix deposition, share many characteristics with skeletal muscle fibroadipocyte progenitors (FAPs) and cancer-associated fibroblasts, which can be derived from ASCs ^[8]^. In both mice and humans, *Dpp4* has been identified as a marker useful for ASC classification ^[8]^, with *Dpp4*^+^ FAPs giving rise to *Dpp4*^−^ preadipocytes ^[5]^.

There are notable age-related changes in the function of preadipocytes and FAPs that have been discovered. In iWAT, a small subpopulation of stromal cells resembling preadipocytes appears early in the post-natal period. This CD142^+^ subpopulation, termed Aregs, lacks inherent adipogenic capacity and can exert an anti-adipogenic effect on ASCs in vitro and upon transplantation in vivo ^[9]^. Another aging-related stromal cell subpopulation, called aging-dependent regulatory cells (ARCs), has also been identified in iWAT of aging mice ^[10]^. ARCs secrete CCL6 and other cytokines that inhibit differentiation and proliferation of adjacent APCs. These cells may contribute to the well-documented reduction of SAT mass and plasticity observed in human aging. Similarly, ASCs from gWAT of aging mice were found to inhibit preadipocyte differentiation through secreted factors. This is likely due to ASCs becoming senescent or at least acquiring features of senescent cells, such as the secretion of SASP factors causing tissue dysfunction ^[11]^.

## 3. Mechanisms of adipocyte progenitor cell proliferation

Understanding basic mechanisms driving APC proliferation is important for developing mechanisms by which to delay premature senescence, as proliferation can ultimately lead to telomere attrition and senescence in stressed cells (reviewed in the study by Huang et al ^[12]^). This is particularly important in the context of aging or overnutrition, both of which lead to the exhaustion of APCs in AT ^[13]^. In humans, APC proliferation and adipogenesis are thought to decline throughout aging ^[14]^. Concomitantly, fat mass decreases with age, leading to increasing lipid storage in tissues other than fat. Studies in rodents support the association of impaired APC renewal and insulin resistance ^[15,16]^.

Multiple factors have been shown to control the proliferation of APCs in vivo, many of which function as endocrine or paracrine signaling factors. For example, fibroblast growth factor 6 (*Fgf6*), a paracrine factor expressed in mature adipocytes, promotes the proliferation of APCs, and continued administration of FGF6 in iWAT can induce proliferation of Pdgfrα^+^ APCs, promote beige fat biogenesis, and increase AT metabolism in the context of high-fat diet (HFD) feeding ^[17]^. *Transforming growth factor-β3 (Tgfβ3*), the expression of which was identified in humans as a factor influencing adipocyte number, can also promote progenitor proliferation. Indeed, *Tgfβ3* haploinsufficiency in mice results in reduced progenitor proliferation, adipocyte hypertrophy in subcutaneous AT, and glucose intolerance in the context of HFD feeding ^[18]^. Glucocorticoids and other adipogenic hormones are secreted in a circadian manner, and adipogenesis is tightly controlled by the pulsatile 24-hour rhythms in glucocorticoid release. Flattening of glucocorticoid rhythms leads to adipocyte hypertrophy and enlargement of fat mass ^[19]^, with an inability of preadipocytes to maintain suppressed adipogenic potential. Among APCs, DPP4-expressing APCs are particularly significant due to their multipotent capabilities and high proliferation rates. Knockdown of this transmembrane glycoprotein greatly reduces self-renewal capacity and proliferation but increases adipogenic potential ^[20]^.

Our group and others have recently discovered the importance of the circadian clock in the regulation of progenitor proliferation. The circadian clock, governed by a cellular transcriptional-translational feedback, aligns metabolic processes across tissues with environmental cues, promoting metabolic coordination across tissues to prevent metabolic diseases ^[21]^. Research has demonstrated that disturbances in circadian rhythms, such as those from chronic jet lag or shift work, can desynchronize cellular clocks, leading to changes in gene expression and increased cell proliferation ^[22]^. Such circadian desynchrony is thought to contribute to increased adiposity, body mass index, and metabolic disease in individuals subjected to chronic circadian disruption ^[23–30]^.

Human subcutaneous fat APCs were shown to harbor circadian rhythmicity over 15 years ago through serum shock experiments in culture ^[31]^. In preclinical studies, transgenic circadian period 2-luciferase (PER2:LUC) reporter mice ^[32]^ and similar transgenic mouse models generated to visualize circadian-driven reporter activity ^[33]^ have been instrumental in demonstrating that primary APCs have functional circadian clocks. Though PER2::LUC reporter mice show rhythms in APCs and mature adipocytes alike, rhythmicity is of a much greater amplitude in APCs compared to mature adipocytes ^[34]^. In APCs, the *period* (*Per*) genes appear to be particularly important for adipogenesis. For example, *period 2* (*Per2*) directly blocks PPARG recruitment to target promoters and loss of *Per2* results in increased adipogenesis ^[35]^. The *Period 3* (*Per3*) gene is also important in adipogenesis and regulates Krüppel-like factor Klf15 to regulate adipogenesis, revealing that circadian mechanisms contribute to the differentiation of APCs, coordinating it with other physiological signals ^[36]^. Like *Per2*, loss of *Per3* increases adipogenesis. Deletion of *Cry1*, part of the so-called “negative arm of the cellular clock”, in 3T3L1 cells, also impairs adipogenesis in vitro ^[37]^.

Five-ethyl-2’-deoxyuridine (EdU) injection experiments, wherein dividing cells can be labeled in vivo for subsequent analysis of cell division, reveal that rhythmicity in the proliferation of progenitors occurs in both VAT and SAT, with a subset of progenitor cells proliferating at higher levels at the end of the feeding phase relative to the end of the fasting phase ^[38]^. The extent to which this attribute of progenitor proliferation is cell autonomous vs non-autonomous is not fully understood and likely depends on endocrine and paracrine factors. However, diurnal waves of proliferation in a subset of APCs are at least partly controlled by diurnal patterns of energy intake, as disruption of diurnal feeding patterns by either fasting or by isocaloric feeding across the light and dark cycle destroys rhythmicity in progenitor proliferation. Loss of the circadian transcription factor, CLOCK, in mice results in an upregulation of genes promoting proliferation in both iWAT and eWAT ^[38]^. Interestingly, loss of the CLOCK interacting protein, Brain and Muscle ARNTL-like protein (BMAL1) in *Pdgfrα*-positive cells in vivo, protects from diet-induced hypertrophy and liver steatosis ^[39]^, likely due to the role of BMAL1 in adipogenesis ^[40]^.

Both timing and quality of nutritional input are important for the circadian regulation of APCs. Overnutrition in the form of HFD feeding is known to drive elevated levels of progenitor cell proliferation in mice ^[38,41,42]^. Our group has shown that HFD feeding derails the rhythmic pattern of progenitor proliferation over the course of the 24-hour cycle, resulting in constitutively high levels of proliferation throughout the 24-hour cycle ^[38,41]^. This effect on the APC clock appears to be long-lasting, as temporary diet reversal is insufficient to restore rhythmic proliferation, though overall levels of proliferation decrease ^[38]^.

In conclusion, ligand-activated receptor signaling pathways and the circadian clock are crucial for regulating APC proliferation and adipogenesis, ensuring optimal AT function in response to both internal and external factors. This complex regulation emphasizes the importance of circadian rhythms for metabolic health and offers insights into potential therapeutic targets for metabolic diseases.

## 4. Subpopulations and proliferation of APCs in brown adipose tissue

Unlike WAT, which can undergo pathogenesis underlying metabolic diseases, brown adipose tissue (BAT) ^[43–45]^ supports healthy metabolism ^[46,47]^. Brown adipocytes are abundant in rodents ^[48]^ and can be activated in humans ^[49–52]^. Brown adipogenesis is induced by cold exposure, exercise, and other sympathetic nervous system (SNS) stimuli that act via catecholamines activating β-adrenergic receptors ^[53–55]^ and, hence, promoting mitochondrial biogenesis ^[56]^. Brown-like (beige) adipocytes, inducible in SAT (and less so in VAT) by the SNS, are functionally similar to BAT adipocytes ^[57–59]^. SNS stimulation activates WAT lipolysis, which makes fatty acids available for BAT non-shivering thermogenesis ^[60]^ enabled by uncoupling protein 1 (UCP1), which disables ATP synthesis during electron transport in mitochondria ^[56,61]^. By increasing the expenditure of energy, dissipated as heat, BAT and beige SAT normalize glucose homeostasis ^[62–65]^. Positive metabolic effects of AT “beiging” in animal models have suggested that activation of adaptive thermogenesis can be used to counter type 2 diabetes ^[66–68]^.

In rodents, adapting to cold temperatures requires the activity of existing brown adipocytes in BAT and the formation of new brown adipocytes from resident APCs. scRNA-seq of BAT stromal vascular cells from mice exposed to cold for 2 or 7 days, combined with cell lineage trajectory analysis, identified vascular smooth muscle cells (VSMCs) expressing transmembrane receptor potential vanilloid 1 (TRPV1) as potential progenitors for cold-induced brown adipocytes ^[69]^. Lineage tracing using the *Trpv1-Cre* line supported this finding, showing that genetically marked *Trpv1*-lineage cells proliferate and differentiate into thermogenic adipocytes upon cold exposure. The specific expression of TRPV1 in BAT’s VSMCs supports the idea of a mural cell origin for adipocytes ^[70,71]^. Independent single-cell studies of thermogenic adipose depots reveal that various stromal subpopulations can give rise to thermogenic adipocytes. Specifically, scRNA-seq analysis of BAT stromal vascular cells after cold exposure identified multiple fibroblastic *Pdgfrα*-expressing subpopulations with distinct tissue localizations ^[72]^. One of these subpopulations, proliferating upon cold exposure, is distinct from *Trpv1*^+^ VSMCs, indicating that multiple progenitor cell populations contribute to cold-induced brown adipogenesis in interscapular brown adipose tissue (iBAT).

Aortic perivascular adipose tissue (PVAT) shares several features with iBAT and contains distinct progenitor cells committed to the brown adipocyte lineage. scRNA-seq analysis of PVAT reveals heterogeneity in brown adipocyte progenitors, showing that VSMCs and various *Pdgfrα*-expressing fibroblastic subpopulations (including committed PPARG-positive preadipocytes and stem-cell-like mesenchymal progenitors) are similar to multipotent cells identified in inguinal white adipose tissue (iWAT) marked by *Pi16* ^[73]^. At this stage, preadipocytes and FAPs, but not VSMCs, can differentiate into brown adipocytes in vitro. These progenitor subpopulations are also present in adult mouse aortic PVAT, where *Trpv1*^+^ VSMCs can differentiate into brown adipocytes ^[73]^. Lineage tracing using *Myh11-Cre* confirmed that VSMCs help maintain brown adipocyte numbers in adult PVAT. These findings collectively suggest that multiple progenitor populations are induced to proliferate and differentiate to maintain depot-specific AT function. It appears that adipogenic *Pdgfrα*^+^ stromal cells drive tissue formation, while adipogenic VSMCs help maintain tissue homeostasis in adults. Additionally, these results highlight how the adipogenic potential of cell populations can change over time.

## 5. Beige adipocyte progenitor cells

In addition to the characterization of cells fated to become fibroblasts vs adipocytes in WAT, scRNA-seq has been utilized to identify potential beige adipocyte progenitors in the context of iWAT. Single-cell analysis of iWAT has revealed a subset of *Pdgfrα*^*+*^ *Sca1*^*+*^ cells with elevated *Cd81* gene expression (“*Cd81*-High”). CD81-High stromal cells demonstrate a VSMC expression profile, rich in *Acta2* and *Sm22,* and contribute to beige adipogenesis ^[74]^. In comparison to *Cd81*-Low cells, *Cd81*-High cells possess a greater adipogenic potential in vitro and differentiate into beige adipocytes upon transplantation and cold exposure in vivo. While CD81 is elevated in iWAT *Pdgfrα*-positive cells, it also shows high expression in most Sca1-positive cell clusters ^[74]^, and compared to *Sm22* and *Acta2*, is an even more specific marker of beige adipogenesis. CD81 acts as a functional marker of beige progenitor cells and associates with integrins that mediate the integrin-FAK signaling cascade triggered by irisin ^[74]^. CRISPR-mediated inactivation of *Cd81* reduces cold-induced beige adipogenesis. Supporting these preclinical studies, reduced numbers of *Cd81*^+^ cells in human subcutaneous WAT are associated with increased metabolic risk, indicating that the biogenesis of beige adipocytes is essential for systemic metabolic health. *Cd81* expression is prevalent in a significant proportion of iWAT *Pdgfrα*^+^ cells, which are enriched within the *Dpp4*^−^ preadipocyte pool. However, it remains uncertain whether *Cd81*^+^ progenitors can also differentiate into white adipocytes under certain conditions.

In the context of aging, beige adipogenesis is impaired, with aged beige APCs overexpressing *Pdgfrβ* to inhibit beige adipogenesis ^[75]^. Genetically depleting *Pdgfrβ* in adult male mice restores beige adipogenesis by blocking STAT1-induced suppression of interleukin 33 (*Il-33*) expression, which impairs cytokine 2 signaling, reducing the immune cell niche in aging fat. Inhibition of the Pdgfrβ-STAT1 signaling pathway can restore cold-induced immune cell activation and beige adipogenesis ^[75]^.

## 6. Bone marrow adipose tissue progenitor cells

Bone marrow adipose tissue (BMAT) is known for its significant plasticity and heterogeneity. Sequencing data has revealed the diversity of skeletal mesenchymal stromal cells within BMAT. One distinct population of committed preadipocytes within the BMAT niche is a cellular population expressing *Adipoq*, *Pparg*, *Cebpa*, and *Lpl*, but lacking *Plin1* and lipid droplets. These are referred to as marrow adipogenic precursors (MALP) ^[76]^ and are similar to pericytes. MALPs are commonly located within bone marrow capillaries, expressing PDGFRβ and sharing a basement membrane with ECs. They also produce significant angiogenic factors like vascular endothelial growth factor A (VEGFA). Their genetic removal leads to a reduction in overall blood vessel density. Accumulation of marrow adipogenesis in the context of aging and obesity results in increased adipocyte numbers and impaired hematopoietic and bone regeneration ^[77]^. Similar to other AT niches, HFD feeding results in a rapid upregulation of the proliferation of this marrow APC population, suggesting that this particular progenitor might also be at risk for premature senescence in the marrow under conditions of overnutrition and aging. The discovery of MALPs supports the idea that pericyte-like cells play a role in the adipogenic lineage and underscores the diverse functions of adipose progenitors in maintaining tissue homeostasis.

## 7. Cell senescence in adipose tissue

Cell aging, leading to cell senescence, is responsible for tissue changes leading to age-related diseases ^[78]^. Accumulation of senescent cells typically results in the SASP, involving the release of a variety of inflammatory cytokines that function in an autocrine or a paracrine manner reinforcing dysfunction of already senescent cells, but also promoting senescence in neighboring cells ^[12]^. Cyclin-dependent kinase inhibitors (CDKIs), p16INK4A (CDKN2A), and p21CIP1 (CDKN1A), typically used as markers of senescent cells, are also expressed in normal cells exiting the cell cycle. Therefore, they need to be used in combination with senescence-associated β-galactosidase, and markers of DNA damage, for example, T53BP1, γH2AX, H3K9me3, H3K27me3, telomere-associated foci (TAF) formation, and the loss of lamin B1 (LMNB1). Functional readouts include induction of cell oxidative metabolism, survival, and proliferation, measured by markers such as Ki-67 or EdU incorporation. Upregulation of anti-apoptotic pathway markers (eg, PI3K, BCL2L1) and cell surface markers linked with senescence (eg, DPP4, TNFRSF10D, oxidized vimentin, and uPAR) are also informative. Finally, the SASP proteins are read out to determine the impact of senescent cell accumulation ^[79]^.

As in other organs, somatic cells in AT are continuously replaced throughout aging. Without continuous telomere length maintenance, progenitor cell over-proliferation accelerates their replicative senescence. The gene *Tert* codes for the enzyme that lengthens telomeres to prevent chromosome shortening during cell division, as well as protects the cell from genotoxic stress and maintains healthy epigenome, mitochondrial function, and bioenergetics ^[78,80]^. TERT activity persists in stem cells but is turned off in somatic cells, which confers telomere erosion and cell aging. While humans are born with telomeres in the 10–15 kb range, mice (C57BL/6) are born with telomeres >50 kb and continue to express TERT in some somatic cells ^[81]^. Thus, they are resistant to replicative senescence and stem cell depletion ^[82]^. By using mice lacking *Tert* in APCs of *Pdgfrβ*^*+*^ or *Pdgfrα*^*+*^ lineages as “humanized” models of telomere attrition, we have reported that diet-induced obesity accelerates APC replicative senescence, which results in the early onset of AT inflammation, fibrosis, adipocyte hypertrophy, and type 2 diabetes ^[13]^.

**Figure 1.**
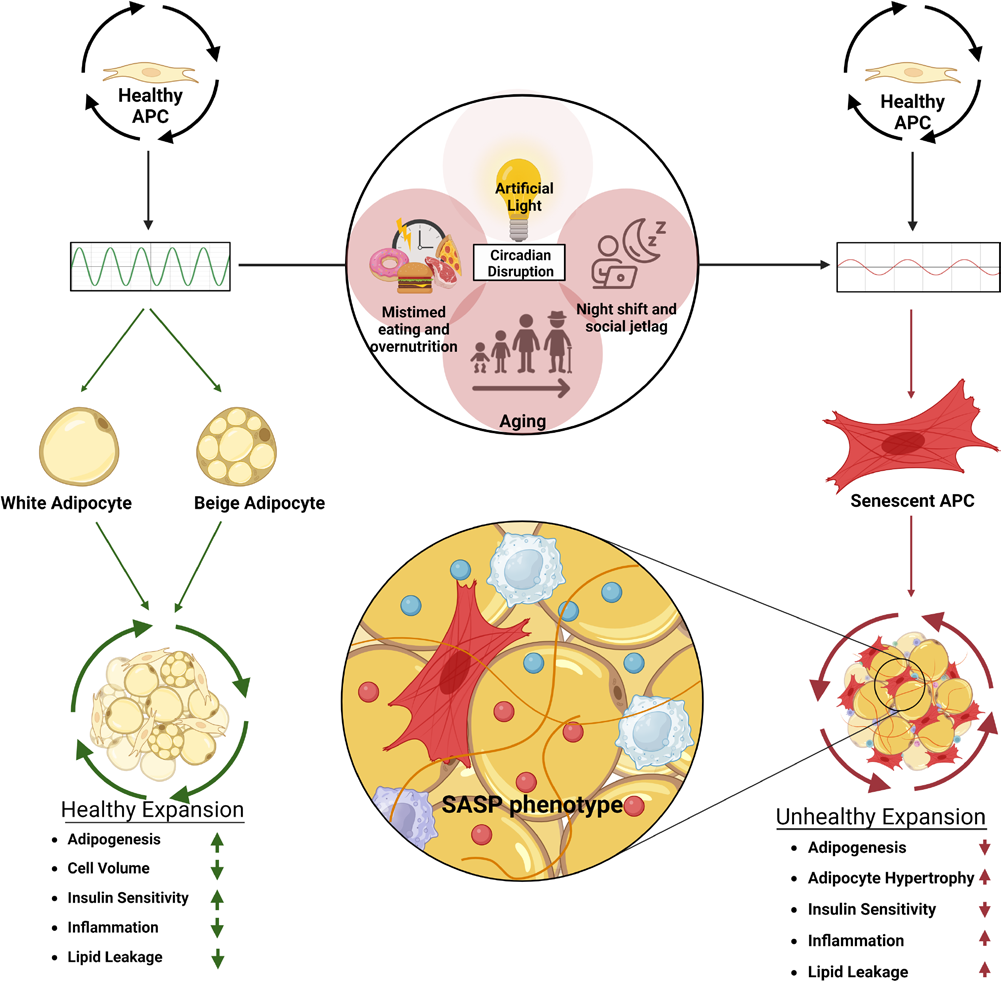
**Environmental factors contributing to elevated and premature senescence of APCs in AT.** Diet and the circadian clock are both important for the proliferation and differentiation of APC into new mature adipocytes capable of storing fatty acids within the AT depot. Overnutrition, aging, and circadian disruption lead to impaired proliferation of APCs and ultimately premature cellular senescence, causing hypertrophy of mature adipocytes, hypoxia, and inflammation within the AT depot. The figure was made using the program BioRender. APC, adipocyte progenitor cell; AT, adipose tissue.

Whether due to replicative senescence or stress-induced senescence, cell senescence in AT is thought to be a primary cause of inflammation. Senescent cells, which release cytokines such as IL6, IL8, and TNFα, can influence neighboring non-senescent cells to also adopt a senescent phenotype ^[83]^. In the context of the fat depot, several consequences of accumulating senescent cells include impaired adipogenesis, adipocyte hypertrophy, and lipid spillover to other tissues (Figure 1) (reviewed in the study by Nerstedt and Smith ^[84]^).

## 8. Intervention of premature senescence in adipose tissue

Due to the impact of cell senescence on AT function and health, finding ways to prevent premature APC senescence and dealing with senescent cells is an active area of investigation. Based on the factors driving APC senescence, several routes to delay early onset proliferation and exhaustion of APCs have been studied.

Proliferation, known to drive telomere attrition over time, is one mechanism by which premature APC senescence can occur. For example, based on data showing that both time and quality of nutrient intake control waves of APC proliferation, a healthier diet devoid of excess calories and timed eating may be one of the approaches to prevent over-proliferation and premature exhaustion of the progenitor pool.

Senolytics, compounds that target the senescent cell anti-apoptotic pathways, show promise in preclinical models in terms of delaying metabolic imbalance caused by a variety of organs and have progressed to clinical trials. In mice, administration of the senolytic combination of a proto-oncogene C-Src (SRC)/tyrosine kinase inhibitor dasatinib and the natural flavonoid quercetin has been reported to suppress inflammation and improve the differentiation capacity of APCs to generate new healthy adipocytes ^[85]^. In a study of humans with diabetic kidney disease, 11 days of senolytic treatment using dasatinib and quercetin resulted in reduced senescence cell accumulation in AT and a reduction of AT macrophage accumulation ^[86]^.

Mouse models based on the inactivation of *Tert* are convenient tools to study senescence intervention approaches. Recently, we have established the mouse model of EC-specific *Tert* knockout ^[87,88]^. We have reported that EC senescence is also linked with telomere-independent mitochondrial dysfunction, increasing vascular permeability and tissue hypoxia ^[87]^. *Tert* knockout ECs have reduced mitochondrial content and function, which results in increased dependence on glycolysis, observed in aging humans. Consistent with this, EC *Tert* knockout mice have glucose intolerance and a decrease in fat tissue mass due to the limitation in vascular expansion. EC *Tert* knockout mice present a “humanized” mouse model of EC aging that can be used for studying mechanisms of aging-associated organ dysfunction. This model of expedited vascular cell senescence is convenient for testing approaches to intervene cells senescence and biological aging.

## 9. Conclusions and future directions

In conclusion, multiple factors control APC proliferation and in turn, adipogenesis to control both AT expansion and capacity to store lipids throughout the lifespan. Elevated proliferation of APCs can expedite their senescence in various AT depots, leading to inflammation and insulin resistance caused by ectopic lipid deposition in organs other than AT. Senescence of APCs in AT inhibits adipogenesis and is a mechanism by which stressors such as overnutrition and circadian disruption exacerbate the aging-associated decline in AT function. Though animal models support the potential for senolytics and *Tert* expression in the regulation of aging-associated senescence, clinical studies will be necessary to determine whether such approaches are also effective in delaying senescence-associated metabolic disease in humans. Certainly, behavioral approaches, such as active phase eating and minimization of circadian disruption throughout the lifespan, are likely to provide optimal protection against senescence-related AT and metabolism dysfunction.

## Conflicts of interest

The authors declare that they have no conflict of interest.

## Funding

This work was supported by NIH grant 5R01DK125922-03 “Metabolic consequences of adipocyte progenitor replicative senescence: mechanism and intervention” to MGK/KEM.
